# Recreation

**Published:** 1907-08

**Authors:** B. S. Searcy

**Affiliations:** Tuscaloosa, Ala.


### RECREATION.

By B. S. Searcy, D. D. S., Tuscaloosa, Ala.

In presenting to you a paper upon this subject it will not be my purpose to attempt to make all of you fisherman, for fishing is hard work, and such that you will not pursue unless you have felt that thrill of delight with which the true fisherman is filled when he sees his cork take a sudden plunge, and, drawing in his line, finds that he has met a match for his angling skill, and only after repeated attempts is able to land his catch.

Neither can I hope to enthuse every one with the desire to become a noted hunter; for sportsmen are born, not made, and only is it necessary to once get a taste, and the instinct to catch and to kill will ever assert itself and seek for chances to be satisfied.

But rather shall I attempt to impress upon you the necessity for some health-preserving recreation, taken systematically each day, as the surest, and I might say the only, way to protect ourselves against the many ills to which mankind has fallen heir.

Yes, truly I believe the subject to be one of more vital importance to the practitioner of dentistry than porcelain fillings and bridges, even so important as our calling itself; for without recreation and diversion no dentist can keep up the requirements forced upon him by the very nature and tediousness of his work.

We are not mechanics that can be so automatically arranged and adjusted as to keep on working for years without a stop and apparently little wear. No, I say, we are not, and I am sure I find an emphatic indorsement in every one present when I say that we all have our limits of endurance; and, although these limits vary a great deal in different men, and at times in the same individual, still it is well to be always on the safer side and not approach too closely that border line, beyond which the sane become insane, the strong man a physical wreck, and the man who once thought he was able to put in seven days each week at work now finds himself so shattered that he is forced to spend a large part of his time traveling from place to place in an attempt to regain that strength that was abused and revolted.

"Mens sana in corpore sano"—a sound mind in a sound body—is the chiefest requisite for the successful dentist to possess to enable him to pursue his calling throughout his allotted time.

It is indeed sad, and I might say discouraging, to see how many of our profession fail. Not so much am I considering those who find that they are not suited to the requirements of the dental profession and soon drop out and seek more congenial vocations, but to the men who have in a few years' time proved their ability to remain in the foremost ranks of the profession; and when their duties and responsibilities have increased to the point where most is demanded of them, there comes disappointment and failure. Some fail because their lungs are not equal to the tax placed upon them; some because their stomachs fail to digest and assimilate the food that should keep the body properly nourished; some become rheumatic; some whose nervous systems prove inadequate to the constant strain upon them and become so shattered as to force them to give up; yes, I might add many other ills that are ever ready to pounce upon the ambitious practitioners, who, sooner or later, are forced to realize that they have attempted to do more than their particular weaknesses would allow, and are sent away by their physicians to rest and recuperate; but often it is too late to regain what should never have been developed to such an alarming extent.

Therefore, let me urge upon one and all to watch yourselves carefully, and do not abuse your greatest blessing, good health, or imperfect health, if such be your state. Guard it, and guard it continually. You may claim that you are too busy and cannot afford to lose the time, as your practice would be injured if you were absent from your office occasionally, to all of which I would answer that keeping at your work without sufficient recreation is but "killing the hen that lays the golden egg."

All are not constituted alike, and to attempt to say how often or how much diversion would be necessary to keep one evenly balanced, to keep his body sound and his nerves steady, would be impossible for any one; but you yourselves are the best judges. Your work will tell you, your friends will tell, your wife will tell you; and should you be so unfortunate as to be overworked and not possess a wife to help you care for yourself and interests, indeed you must be an object of pity!

Get out into the sunshine and fresh air, and get out often; and, in the language of the small boy, "Get out and sweat, and sweat like a horse;" for a short while thus spent each day will surely be like "bread upon the waters," like the "stitch in time." A brisk walk, a few miles of horseback riding, a drive into the country, a bicycle ride, and an automobile ride are all good, and some claim that wood chopping and a short while spent working in the garden are hard to beat as health giving exercises.

The ways are innumerable, and the one or several that you will the more regularly pursue should be adopted; nor should you confine yourself to any one way; but have only one rule, and let that be to never let a day pass without having spent a part of it exercising as a preservative of your health and protective measure against the inroads of disease, and thus to so blend your work and your pleasure as to make it a pleasure to work.

It is told that a man of our town once wished that he was worth forty thousand dollars, for then he would consider himself sufficiently well off to place this notice upon the door of his small grocery store as often as he wished: "Go to hell; gone fishing." I would not advise wording it just that way, for fear some friend might change it somewhat and have it read: "Gone to hell; going fishing." But still put one there occasionally; it might be "Out of Town;" for you know not how the trout will bite upon that other shore, or whether there will be any bird shooting allowed over there.

When spring comes round and you begin to feel the depressing effect of the warm weather, there is no better time to take off a few days from your labors to better prepare yourself for the siege of the summer days, and no more profitable place than the meeting of the State Dental Association, there seeing and hearing what others are doing toward making the dental profession what it is today; and if you have only a penny's worth of information that will help lift it even that little bit higher, place it there where it will help to make thy brother's labor less ardous and thy fellow-man's burden less heavy.

